# Pharmacological inhibition of fatty acid-binding protein 4 (FABP4) protects against renal ischemia-reperfusion injury

**DOI:** 10.1039/c8ra00122g

**Published:** 2018-04-23

**Authors:** Min Shi, Rongshuang Huang, Fan Guo, Lingzhi Li, Yanhuan Feng, Zhengjie Wei, Li Zhou, Liang Ma, Ping Fu

**Affiliations:** Kidney Research Institute, Division of Nephrology, West China Hospital of Sichuan University Chengdu 610041 No. 37 Guoxue Allay China Liang_m@scu.edu.cn fupinghx@163.com +86-28-85423341 +86-28-85164167; Chengdu No. 7 High School Chengdu 610041 China

## Abstract

Fatty acid-binding protein 4 (FABP4) is a key mediator of endoplasmic reticulum (ER) stress and apoptosis in diabetes and atherosclerosis. Studies also confirmed that circulating FABP4 depended on renal function in chronic kidney disease (CKD) and acute kidney injury (AKI) patients. However, the function of FABP4 in AKI remains poorly understood and the aim of this study was to investigate the role of FABP4 in ischemia-reperfusion (I/R)-induced AKI. In the present study, renal I/R injury triggered the high expression of the FABP4 gene and protein in the nucleus and cytoplasm of tubular cells of mouse kidney tissue compared to that of Sham. Pretreatment with BMS309403, a highly selective inhibitor of FABP4 at a dose of 20 mg kg^−1^ d^−1^ for 4 d, significantly reduced serum creatinine levels to improve acute renal dysfunction and attenuated renal tubular damage in injured kidneys. Pharmacological inhibition of FABP4 also decreased the number of TdT-mediated dUTP nick-end labeling (TUNEL) positive apoptotic tubular cells, accompanied by the down-regulation of cleaved-caspase-3 expression. Furthermore, oral administration of FABP4 inhibitor resulted in a significant attenuation of ER stress indicated by its maker proteins expression of glucose-regulated protein 78 (GRP78), C/EBP homologous protein (CHOP), and caspase-12 in I/R injured kidneys. *In vitro*, the increased expression of FABP4 in the human renal proximal tubule cell line (HK-2 cell) was induced by hypoxia followed by reoxygenation (HR) and the FABP4 inhibitor resulted in a significant attenuation of cell apoptosis and ER stress in HR-induced HK-2 cells. In summary, these findings indicated that FABP4 contributed to the pathogenesis of I/R-induced AKI and suggested that the inhibition of FABP4 might be a promising therapeutic strategy for AKI treatment.

## Abstract 2

FABP4 inhibition might attenuate I/R-induced AKI through reducing ER stress and apoptosis.

## Graphical abstract

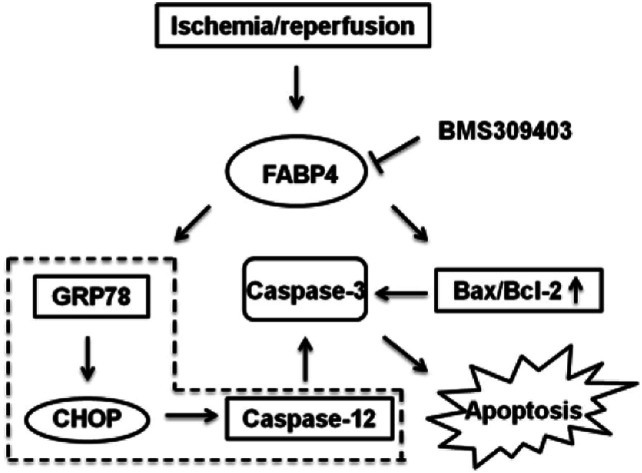

## Introduction

Acute kidney injury (AKI) is a growing public health burden. The incidence of AKI steadily rises, leading to high morbidity, high mortality, and increased health care costs through hospitalizations and development of chronic kidney disease (CKD).^1–4^ Renal ischemia-reperfusion (I/R) injury is the most common cause of AKI, and the incidence of AKI exceeds 50% after major cardiac, aortic, or transplant surgeries.^5,6^ Although the pathogenesis of renal I/R injury is complicated, decades of studies have gained significant insights into the mechanisms of ischemic AKI, including tubular cell injury and death, vascular damage and inflammatory response, *etc.*^7–10^ During these pathological processes, kidney hypoxia and subsequent re-oxygenation induces tubular cells apoptosis *via* endoplasmic reticulum (ER) stress pathway, which is a major event in renal I/R-induced AKI.^11,12^ However, there is still lacking of approved therapy for the treatment of AKI.

Fatty acid-binding protein 4 (FABP4), also known as adipocyte fatty acid-binding protein (AFABP) or adipocyte protein 2 (aP2), is a lipid-binding chaperone highly expressed in adipocytes and macrophages.^13,14^ It has been demonstrated that mitigation of ER stress and apoptosis is protective against atherosclerosis and FABP4 was an obligatory intermediate for macrophage ER stress responses to lipids.^15–18^ In addition, chemical or genetic inhibition of FABP4 could improve atherosclerosis and type 2 diabetes mellitus, and targeting FABP4 offers therapeutic approaches for several inflammatory and metabolic diseases.^19,20^

Previous studies also confirmed that circulating FABP4 depended on renal function in CKD and AKI patients.^21,22^ Urinary FABP4 level also could predict yearly decline of renal function and would be a new marker of kidney damage.^23^ Other than macrophages and adipocytes, it has been reported that FABP4 is expressed in the glomerulus and tubular cells in patients with CKD.^24^ Moreover, FABP4 expression in the kidneys induced by CKD is closely associated with renal dysfunction.^24^

**Fig. 1:**
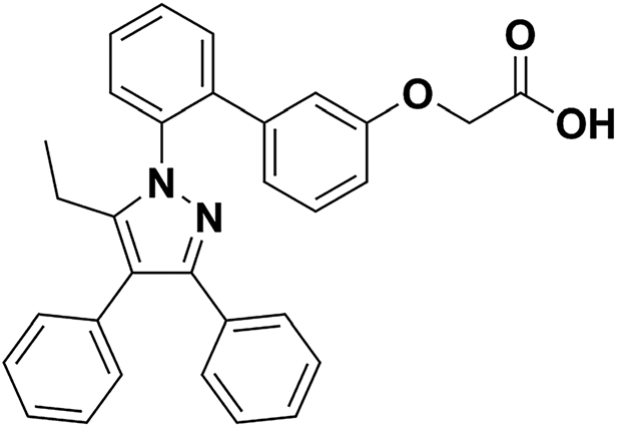
The chemical structure of BMS309403.

However, the role and mechanisms of FABP4 in I/R-induced AKI remained poorly understood. In the study, we evaluated the pharmacological inhibition of FABP4 by a highly selective inhibitor BMS309403 (Fig. 1) on I/R-induced AKI and the mechanisms involved.

## Results

### Renal I/R injury induced the high expression of FABP4 in kidney

**Fig. 2:**
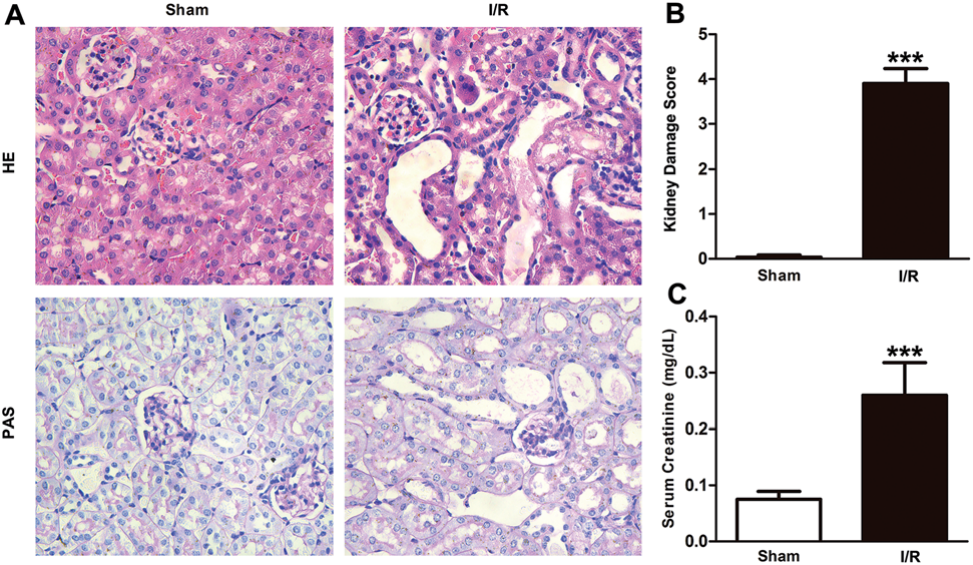
Histological findings of HE and PAS (A), kidney damage score (B) and levels of SCr (C) in mice. HE/PAS-stained sections showed no damage in Sham group and histological damage in I/R group (original magnification × 400). Data were expressed as means ± SD for groups of 6 mice. ***p < 0.001 *vs.* Sham group.

Firstly, renal surgery by 30 min of ischemia and reperfusion of 24 h successfully induced AKI in 10–12 weeks old mice. Compared to Sham mice, renal tubular injury was evident in the cortex of I/R mice (Fig. 2A), with severe dilation of the proximal tubules, cast formation, and massive detachment and necrosis of the tubular epithelium. The tubular injury score of kidney sections and kidney function indicated by serum creatinine (SCr) level showed significant increases in I/R mice (Fig. 2B and C).

**Fig. 3:**
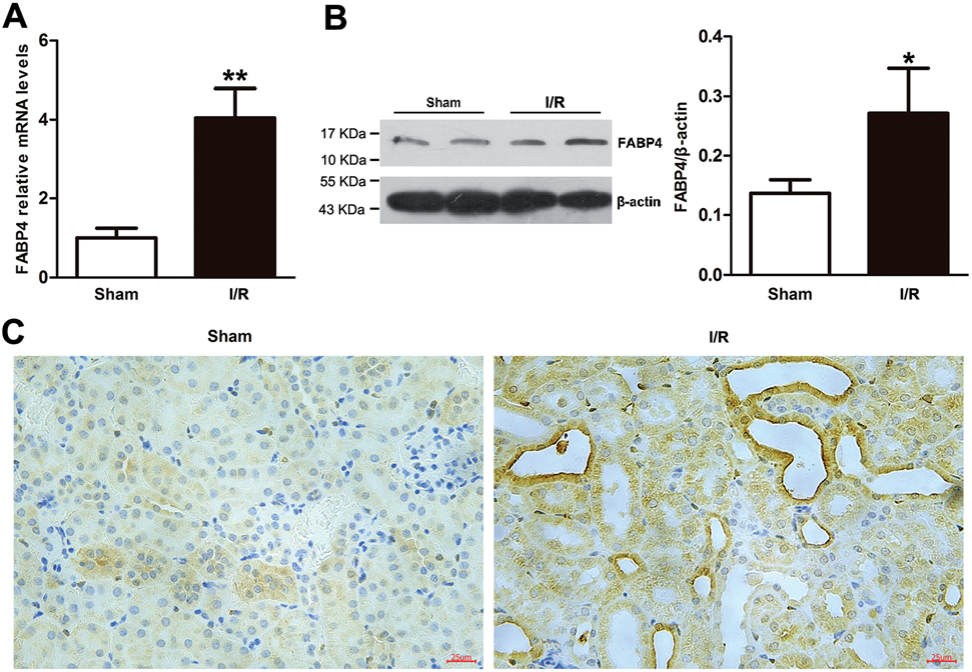
Renal expression of FABP4 was induced by renal I/R injury. (A) FABP4 mRNA expression in renal tissue was measured by real-time PCR. (B) FABP4 protein expression in renal tissue was determined by western blot analysis. (C) The immunohistochemical staining of FABP4 showed FABP4 was markedly induced in the tubular epithelial cells (original magnification × 400). Data were expressed as means ± SD for groups of 6 mice. *p < 0.05 and **p < 0.01 *vs.* Sham group.

**Fig. 4:**
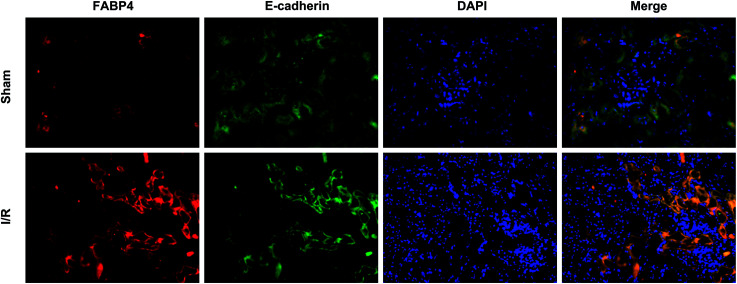
Representative double immunofluorescence labeling images of mice kidneys for FABP4 (red) and E-cadherin (green). DAPI (blue) indicates nuclear stain. There were increased positive signals of FABP4 (red) existing in tubular epithelial cells (green) of renal I/R mice (original magnification × 400).

Recent studies have reported that FABP4 is expressed in the glomerulus and tubular cells in CKD patients.^24^ To investigate whether FABP4 in kidney was induced by AKI, we measured renal expression of FABP4 in a mouse model of I/R injury. As shown in Fig. 3A, renal I/R injury resulted in a significant induction of FABP4 mRNA expression. The I/R induction of FABP4 was also confirmed at the protein level by western blot analysis (Fig. 3B). Immunohistochemical staining of renal tissue revealed that FABP4 was expressed at a low level in macrophages as well as tubular cells of Sham mice (Fig. 3C). Expression of FABP4 in tubular epithelial cells was markedly increased in the mice subjected to renal I/R surgery (Fig. 3C). FABP4 was found to be expressed in both the nucleus and cytoplasm in renal tubular cells. Then the localization of FABP4 in kidney tubular cells was confirmed by double immunofluorescence labeling of E-cadherin (green) and FABP4 (red). Compared with Sham mice, increased positive signals of FABP4 were observed in tubular cells of renal I/R mice (Fig. 4). Taken together, our data illustrated that renal I/R injury induced the high expression of FABP4 in tubular cells of kidneys.

### Pharmacological inhibition of FABP4 attenuated renal I/R-induced AKI

**Fig. 5:**
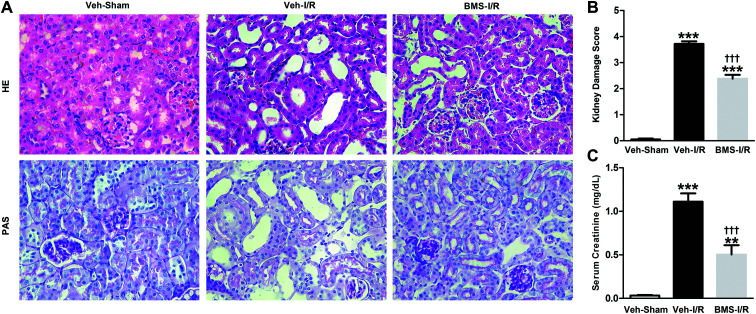
Inhibition of FABP4 with BMS309403 attenuated I/R-induced AKI. (A) HE/PAS staining of the kidney tissues in Sham mice with vehicle or I/R mice with/without BMS309403 (original magnification × 400). (B) Kidney damage score. (C) Levels of SCr. Data were expressed as means ± SD for groups of 6 mice. **p < 0.01 and ***p < 0.001 *vs.* Veh-Sham group; ^†††^*p* < 0.001 *vs.* Veh-I/R group.

As a highly selective small-molecule inhibitor of FABP4, BMS309403 is an orally active agent against atherosclerosis and type 2 diabetes in animal models.^25^ To determine whether inhibiting FABP4 may possess a renal protective effect, we examined the effect of BMS309403 on pathological changes and renal function in I/R-induced AKI. As demonstrated in Fig. 5A, pretreatment with BMS309403 alleviated renal histological injury induced by I/R. Quantification of kidney damage showed that the I/R induced damage score was reduced from 3.7 of vehicle-treated mice to 2.4 of BMS309403-treated mice (Fig. 5B). BMS309403 administration also improved kidney function, which was confirmed by the decreased levels of SCr (Fig. 5C). These data highlighted that pharmacological inhibition of FABP4 alleviated I/R-induced AKI.

### FABP4 inhibitor attenuated tubular cells apoptosis in the kidneys of mice with I/R injury

**Fig. 6:**
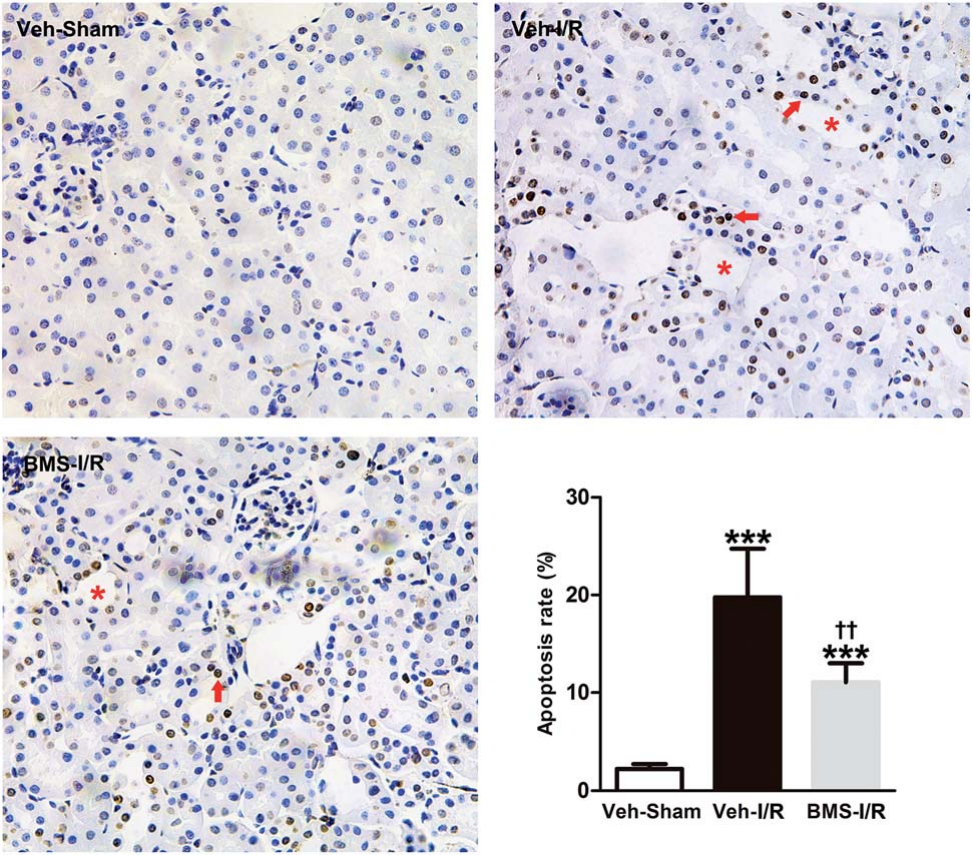
FABP4 inhibitor attenuated tubular cells apoptosis. There were many TUNEL positive apoptotic cells (arrow) in the damaged tubules (asterisk) in renal I/R mice with or without BMS309403 treatment (original magnification × 400). Data were expressed as means ± SD for groups of 6 mice. ***p < 0.001 *vs.* Veh-Sham group; ^††^*p* < 0.01 *vs.* Veh-I/R group.

As tubular cell apoptosis played crucial roles in the pathogenesis of I/R-induced AKI,^11^ we examined the effect of FABP4 inhibition on tubular cell apoptosis following I/R injury by TUNEL staining. As shown in Fig. 6, there were few TUNEL positive cells in the kidneys of Sham mice, whereas many TUNEL positive renal tubular cells were detected in injured kidneys after I/R surgery. Oral administration of FABP4 inhibitor BMS309403 reduced TUNEL-positive cells in the kidneys of I/R injury mice compared with those without BMS309403.

**Fig. 7:**
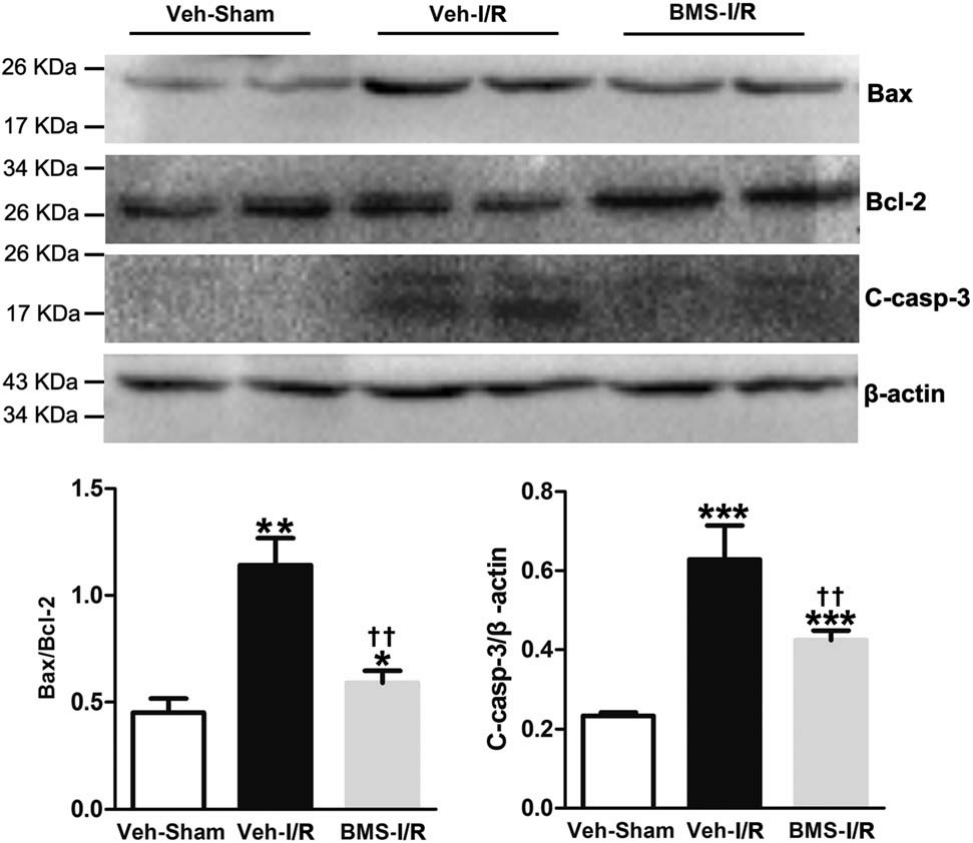
Effect of FABP4 inhibitor on Bax/Bcl-2 and cleaved-caspase-3 levels in injured kidneys of mice. Expression of Bax, Bcl-2 and cleaved-caspase-3 proteins were assessed by western blot analysis. Data expressed as means ± SD for groups of 6 mice. *p < 0.05, **p < 0.01 and ***p < 0.001 *vs.* Veh-Sham group; ^††^*p* < 0.01 *vs.* Veh-I/R group.

I/R injury markedly increased the Bax/Bcl-2 ratio in a proapoptotic direction.^26^ Caspase-3 was a primary mediator in the pathogenesis of I/R-induced tubular cells apoptosis.^9^ Thus, we examined the expression of Bax/Bcl-2 and cleaved-caspase-3 in the I/R-injured kidneys with/without treatment of BMS309403 by western blot analysis. Consistent with TUNEL staining results, the ratio of Bax/Bcl-2 and levels of cleaved-caspase-3 in the kidneys were remarkably increased in I/R mice compared to that of Sham. Significantly, BMS309403 administration reduced the ratio of Bax/Bcl-2 and the expression of cleaved-caspase-3 in kidneys of mice with renal I/R injury (Fig. 7). These data indicated that the inhibition of FABP4 attenuated tubular cell apoptosis in I/R-induced AKI.

### FABP4 inhibitor attenuated renal I/R-induced ER stress

It has been demonstrated that ER stress contributes to I/R-induced AKI initiation and progression.^11^ And FABP4 has been documented to be involved in ER stress in atherosclerosis and diabetic nephropathy.^15,18^ To evaluate if FABP4 participates in AKI-associated ER stress, we further measured expression of ER stress-related proteins in kidneys.

**Fig. 8:**
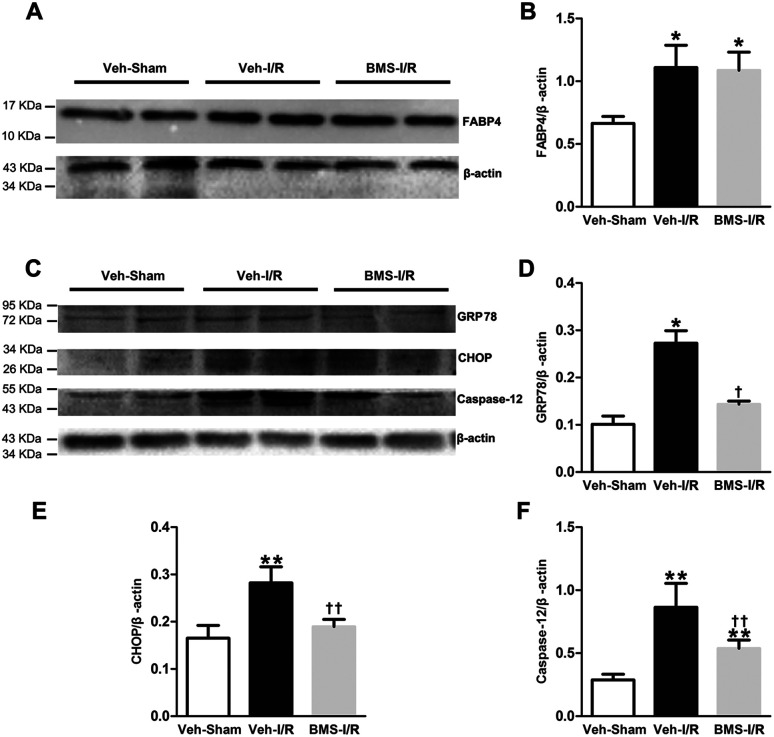
FABP4 inhibitor decreased renal I/R-induced high expression of ER stress-associated proteins. (A) FABP4 expression measured by western blot analysis. (B) Ratio between FABP4 and β-actin measured by western blot analysis. (C) Expression of GRP78, CHOP and caspase-12 proteins were assessed by western blot analysis. (D) Ratio between GRP78 and β-actin. (E) Ratio between CHOP and β-actin. (F) Ratio between caspase-12 and β-actin. Data expressed as means ± SD for groups of 6 mice. *p < 0.05 and **p < 0.01 *vs.* Veh-Sham group; ^†^*p* < 0.05 and ^††^*p* < 0.01 *vs.* Veh-I/R group.

As illustrated in Fig. 8, there was no change in levels of FABP4 in the renal tissue after 4 days of BMS309403 treatment (Fig. 8A and B). Expression of ER stress-associated proteins including GRP78, CHOP and caspase-12 were significantly upregulated in the kidneys after I/R surgery and suppressed in the injured kidneys with BMS309403 administration (Fig. 8C–F).

### FABP4 inhibitor attenuated HR-induced apoptosis and ER stress in HK-2 cells

**Fig. 9:**
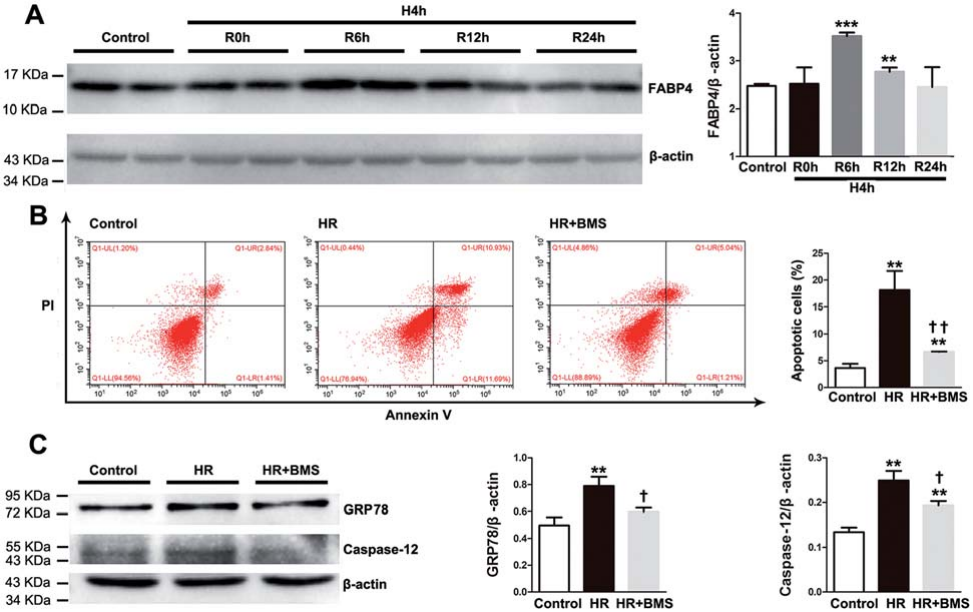
FABP4 inhibitor attenuated HR-induced apoptosis and ER stress in HK-2 cells. (A) FABP4 expression and ration between FABP4 and β-actin in HK-2 cells measured by western blot analysis. (B) Apoptotic HK-2 cells were labelled by annexin V/PI and quantitatively assessed by flow cytometer. (C) Expression of GRP78 and caspase-12 proteins, ratio between GRP78 and β-actin, and ratio between caspase-12 and β-actin were measured by western blot analysis. Data expressed as means ± SD for groups of 3 independent experiments. **p < 0.01 and ***p < 0.001 *vs.* control group; ^†^*p* < 0.05 and ^††^*p* < 0.01 *vs.* HR group.

We also examined the protective effects of FABP4 inhibitor on the renal proximal tubular cell, which is the main site of I/R-induced AKI, using a hypoxia/reoxygenation (HR) injury model *in vitro*. As shown in Fig. 9A, the expression of FABP4 protein in HK-2 cells was significantly increased in 6 h and 12 h reoxygenation after 4 h hypoxia, compared with the non-hypoxic control, with a peak at 6 h.

Based on this result, we performed subsequent experiments under 4 h hypoxia followed by 6 h reoxygenation conditions. Our results showed that HR treatment significantly induced apoptosis in HK-2 cells compared with control (18.14% ± 3.56% *vs.* 3.63% ± 0.80%, *p* < 0.01). FABP4 inhibitor BMS309403 effectively lowered the proportion of apoptotic HK-2 cells induced by HR (6.66% ± 0.06%, *p* < 0.01, Fig. 9B). In addition, expression of ER stress-associated proteins including GRP78 and caspase-12 were significantly upregulated in HK-2 cells after HR and decreased by BMS309403 treatment (Fig. 9C). These *in vitro* study results further indicated the anti-apoptotic and anti-ER stress effects of FABP4 inhibitor on tubular cells.

### Mechanisms of FABP4 inhibition-elicited attenuation of I/R-induced AKI

**Fig. 10:**
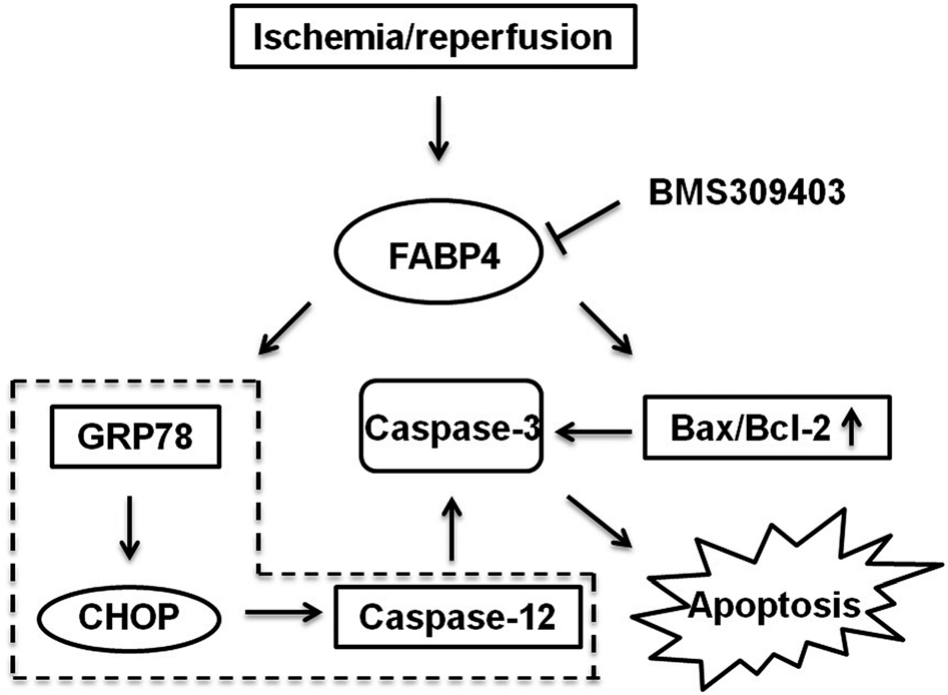
A schematic of mechanisms of renal protection with FABP4 inhibition in renal I/R.

As indicated in Fig. 10, we concluded the mechanisms of FABP4 inhibition-elicited protective role in I/R-induced AKI. Our results highlighted FABP4 inhibition might attenuate AKI through reducing ER stress and apoptosis (Fig. 10).

## Discussion

FABP4 has a well-established role in the development and progression of diabetes and atherosclerosis,^15–17,19,20^ but the effect of FABP4 in AKI has not been determined. Our study reported for the first time that overexpression of FABP4 in kidneys, mainly expressed in tubular cells was induced by AKI after I/R surgery. We also found that the chemical inhibition of FABP4 by BMS309403 resulted in ameliorating renal structural damage and improving renal function in mice models of I/R-induced AKI. Moreover, we demonstrated that the functional inhibition of FABP4 attenuated tubular cells apoptosis and ER stress induced by renal I/R. These findings revealed an important role of FABP4 in the pathogenesis of AKI. Therefore, targeting of FABP4 might be a potential therapeutic strategy for AKI.

Previous studies have reported that FABP4 is detected in some tubular cells in patients with stage IV lupus nephritis, but not in normal kidneys.^24^ The findings suggested that the increase of FABP4 in renal tubular cells might be associated with renal injury. In the present study, FABP4 is remarkably expressed in tubular epithelial cells in mice following renal I/R injury by immunohistochemical staining. These data highlighted that the high expression of FABP4 in tubular epithelial cells was related to I/R-induced AKI. Additional support for I/R injury causing an overexpression of FABP4 in tubular cells could be seen in our immunofluorescence results. In addition, increased expression of FABP4 was induced by HR in HK-2 cells *in vitro*. FABP4 was also expressed in macrophages in Sham and I/R mice as previously reported.^24^ Thus, further studies are needed to determine whether FABP4 expression in macrophages could be increased by I/R injury.

It has been documented that circulating and urinary FABP4 levels are associated with renal dysfunction.^21–23^ Whether the increase in FABP4 promotes renal injury remains unclear. In the study, we chose a highly selective inhibitor of FABP4, BMS309403 to investigate the role of FABP4 in I/R-induced AKI. BMS309403 interacted with the fatty-acid-binding pocket of FABP4 and competitively inhibited the binding of effective molecules. Consistent with previous reports, after 4 days of BMS309403 treatment, there was no significant change in protein level of FABP4 in the injured kidneys.^25^ However, oral administration of BMS309403 attenuated renal histological injuries and improved renal function in mice with renal I/R injury. These data suggested that the inhibition of FABP4 could protect against I/R-induced AKI.

The present data supported previous studies that tubular cell apoptosis was a crucial pathogenic mechanism leading to AKI after I/R surgery,^27^ as noted by the increase of TUNEL positive apoptotic tubular cells in injured kidneys. Furthermore, we found the protein level of cleaved-caspase-3 and Bax/Bcl-2 ratio were significantly increased in the kidneys following renal I/R injury. It has been demonstrated that ischemia markedly increased the Bax/Bcl-2 ratio in a proapoptotic direction and caspase-3 activation was the predominant mechanism underlying the pathogenesis of I/R-induced tubular cells apoptosis.^9,26^ These data further confirmed that renal tubular epithelial cell apoptosis induced by I/R injury played a key role in the development and progression of AKI. However, FABP4 inhibition remarkably decreased the number of TUNEL positive apoptotic cells as well as the elevated expression of cleaved-caspase-3 and Bax/Bcl-2 in the injured kidneys and decreased the percentage of apoptotic HK-2 cells in the study. Collectively, these data suggested that inhibition of FABP4 prevented tubular epithelia apoptosis in renal I/R injury.

ER stress, an initiator of tubular epithelial injury following renal I/R injury, could be triggered by ischemia, and then activated the intracellular signal transduction pathway, which was called the unfolded protein response (UPR).^12,28^ GRP78, a molecular chaperone in cells, served as a master modulator for the UPR network.^15^ Under ER stress, UPR triggered apoptosis through its proapoptotic branches, CHOP and caspase-12.^29^ Therefore, GRP78, CHOP and caspase-12 were considered as key markers of the ER stress level. In our study, we found that along with up-regulation of FABP4, the protein levels of GRP78, CHOP, caspase-12 in I/R injured kidneys of mice and HR-treated HK-2 cells were increased, whereas FABP4 inhibitor BMS309403 decreased the levels of corresponding proteins. These data indicated that protective effects of BMS309403 against AKI were mediated, at least partially, by the inhibition of renal ER stress. Further studies confirmed ER stress was a major factor and played an important role in I/R-induced tubular cells apoptosis.^11,12,30^ Caspase-12, was an ER-specific caspase, which helped trigger ER stress-apoptosis through a caspase-9/caspase-3 cascade reaction.^31^ Our data showed FABP4 inhibition decreased the expression of caspase-12 and activated caspase-3, suggesting that FABP4 inhibitor's anti-apoptotic effect might be partially attributable to suppression of ER stress. Further studies are required to determine whether FABP4 inhibitor suppress tubular cell apoptosis through the reduction of ER stress response.

## Experimental

### Animal models of I/R-induced AKI

The animal experiments were conducted in accordance with a protocol approved by Animal Care and Use Ethics Committee of Sichuan University in China (IACUC number: 2017080A). Male C57BL/6 mice (10–12 weeks old, weighing 25–30 g) were purchased from Animal Laboratory Center of Sichuan University (Chengdu, China). The 12 mice were randomly divided into two groups: Sham and I/R group. After anaesthetized, a midline laparotomy was performed with minimal dissection and both kidneys of mouse were exposed. Renal I/R injury was induced by clamping renal pedicles with non-traumatic microvascular clamps for 30 min, followed by 24 h reperfusion after clamp removal. Occlusion and reperfusion were confirmed by changes in the color of the kidneys. Sham group mice underwent the same surgical procedures but without pedicle clamping.

Further, 18 mice were randomly divided into three groups: Veh-Sham, Veh-I/R and BMS-I/R group. The mice of BMS-I/R were pretreated with a gavage of FABP4 inhibitor BMS309403 (dissolved in 200 μL 30% PEG400) at a dose of 20 mg kg^−1^ d^−1^ and the Veh-I/R was given to the same volume of 30% PEG400 in saline for 4 consecutive days before I/R surgery. The mice of Veh-Sham group were treated with vehicle for 4 days before a Sham procedure.

### Renal histological examination

Kidney tissues were fixed in 10% neutral buffered formalin, embedded in paraffin, and sectioned at 4 μm thickness. After deparaffinization and rehydration, kidney sections were stained with hematoxylinandeosin (HE) or periodic acid-Schiff (PAS). Renal histological damage was examined in a blinded manner and scored according to previous described 0–4 semiquantitative scale.^32^ Briefly, ten fields of cortical tissues per mouse were counted, and the percentage of death area in which tubular dilation, epithelial necrosis and intratubular cast formation was determined, in which 0, no damage; (1) <25%; (2) 25–50%; (3) 50–75%; (4) >75%.

### Measurement of renal function

Renal function was evaluated by serum creatinine, which was determined by HPLC-MS/MS method.

### Quantitative real-time PCR analysis

Total RNA from renal tissues was extracted using a total RNA extraction Kit (Biotek, Winooski, VT, USA) according to the protocols. The concentration of mRNA was tested using a Scan Drop 100 (Analytik Jena, Thuringia, Germany) determiner. Quantitative real-time PCR was performed after reverse transcription by using the fast qPCR kit (Kapa Biosystems, Foster, CA, USA) in a PCR system (CFX Connect; Bio-Rad, Hercules, CA, USA). Relative expression levels of FABP4 were normalized to β-actin. The primer sequences were: FABP4 – forward, 5′-AAACACCGAGATTTCCTT-3′ reverse, 5′-TTATGATGCTCTTCACCTT-3′; β-actin - forward, 5′-TATGGAATCCTGTGGCATC-3′ reverse, 5′-GTGTTGGCATAGAGGTCTT-3′.

### Western blot analysis

Proteins were extracted from kidney tissues or HK-2 cells using RIPA buffer containing 4% cocktail proteinase inhibitors and then analyzed by western blotting. Equal amounts of protein were separated by SDS-polycrylamide gels and then transferred onto a PVDF membrane (Bio-Rad, Hercules, CA, USA). The membranes were incubated with primary antibodies against FABP4 (Abcam, Cambridge, MA, USA), Bax (Abcam, Cambridge, MA, USA), Bcl-2 (Abcam, Cambridge, MA, USA), cleaved-casp-3 (Cell Signaling Technology), GRP78 (Cell Signaling Technology), CHOP (Cell Signaling Technology), caspase-12 (Cell Signaling Technology, Beverly, MA, USA) and β-actin (Abcam, Cambridge, MA, USA) overnight at 4 °C followed by incubation with secondary antibodies (R&D Systems, MI, USA) for 1 h at room temperature. Finally, the proteins were developed with an enhanced chemiluminescence agent (Millipore Corporation, Boston, MA, USA). The signals were measured using an Odyssey Infrared Imaging System (Bio-Rad, ChemiDoc MP, mANUSC, Bio-Rad Laboratories Inc., Hercules, CA, USA) and quantified using the Image J program (National Institutes of Health, Bethesda, MD, USA). The ratio for the target proteins were normalized against β-actin.

### Immunohistochemistry and double immunofluorescence labeling

Heat-induced epitope retrieval was performed on dewaxed sections in citrate buffer (pH 6.0) at 95 °C for 40 min. Endogenous peroxidase activity of tissues was quenched with 3% H_2_O_2_. Then slides were incubated with primary antibody, anti-FABP4 antibody (Abcam, Cambridge, MA, USA) diluted to 1 : 250 in PBS in a humidified chamber overnight at 4 °C. After washing, slides were incubated with horseradish peroxidase (HRP)-incubated secondary antibody (Abcam, Cambridge, MA, USA) for 45 min. After washing, the immunoreactivity of slides was revealed by diaminobenzidine (DAB). Nuclear staining was performed with hematoxylin. The slides were observed under a light microscope.

For double immunofluorescence labeling assay, the slides were incubated with anti-FABP4 antibody (1 : 250) and anti-E-cadherin antibody (1 : 100) overnight at 4 °C and then with fluorescent-conjugated secondary antibodies for 1 h at room temperature. The DAPI (4′, 6-diamidino-2-phenylindole) staining was conducted according to the experimental protocol. Fluorescent specimens were examined using a fluorescence microscope (Olympus).

### Cell culture and HR treatment

Human renal proximal tubule cell line (HK-2 cell) was a gift from Prof. Xueqing, Yu (The first Affiliated Hospital, Sun Yat-Sen University) and maintained in Dulbecco's modified Eagle's medium (DMEM)/F12 (Hyclone, Beijing, China) supplemented with 10% fetal bovine serum (FBS, Hyclone, Australia) at 37 °C under humidified atmosphere of 5% CO_2_ and 95% air.

HK-2 cells were cultured under hypoxic conditions (5% CO_2_, 1% O_2,_ and 94% N_2_) in FBS-free medium for 4 hours at 37 °C to induce hypoxic injury; then, the cells were cultured in 5% CO_2_ and 95% air for reoxygenation. The cells were harvested at 0, 6, 12, or 24 hours after reoxygenation. The groups were randomly divided into the following groups: (1) control: HK-2 cells were incubated in normoxic condition (5% CO_2_ and 95% air) without BMS309403 treatment. (2) HR : HK-2 cells were exposed to 4 h of hypoxia (5% CO_2_, 1% O_2_, and 94% N_2_) followed by 6 h of reoxygenation (5% CO_2_ and 95% air). (3) HR + BMS : HK-2 cells treated with BMS309403 (10 μmol L^−1^) were exposed to 4 h of hypoxia followed by 6 h of reoxygenation.

### Apoptosis detection

*In vivo*, the terminal deoxynucleotidyl transferase-mediated dUTP nick end labeling (TUNEL) staining was conducted on paraffin-embedded slides using an *in situ* cell death detection kit (Roche Applied Science) according to the experimental protocol. The number of TUNEL positive cells in the cortex was counted in five random fields of view on each sample under high-power fields in a blinded fashion. Data were expressed as the ratio of the number of TUNEL positive cells to that of total cells.

*In vitro*, HR induced HK-2 cells apoptosis was measured by flow cytometer using the FITC Annexin V Apoptosis Detection Kit I (BD Biosciences, Indianapolis, IN, USA) according to the instructions. Briefly, HK-2 cells were harvested after experimental treatments *via* centrifugation at 1200 rpm for 3 min and washed twice by PBS. HK-2 cells were resuspended in a 300 μL binding buffer, stained with annexin V-FITC solution (3 μL) for 10 min and then stained with a propidium iodide (PI) (3 uL) solution for 5 min in the dark. Cells (20 000 cells for each sample) were analyzed by flow cytometer (Beckman Cytoflex, Beckman Coulter Australia Pty Ltd., Lane Cove, NSW, Australia). The percentage of early apoptotic (Annexin V + PI−) and late apoptotic (Annexin V + PI+) cells were calculated in total.

### Statistical analysis

Results were analyzed using the SPSS software version 16.0 (SPSS Inc., Chicago, IL, USA). Data were expressed as means ± standard deviation (SD). One-way analysis of variance (ANOVA) followed by the Student-Newman-Keuls (SNK) test were used for multiple group comparisons. Comparisons between two groups were performed by the two-tailed *t* test. The ordinal values of the kidney damage scores were analyzed by Mann–Whitney nonparametric test or the Kruskal–Wallis ANOVA on ranks followed by Fisher exacts test. The level *p* < 0.05 was considered statistically significant.

## Conclusions

In summary, our study demonstrated for the first time that FABP4 played a crucial role in the pathogenesis of I/R-induced AKI. Inhibition of FABP4 with BMS309403 attenuated pathological changes and improved renal function following I/R injury. The mechanisms involved in the suppression of apoptosis of tubular cells and inhibiting ER stress. Thus, selective inhibition of FABP4 could be a potential therapeutic approach for the treatment of AKI.

## Contributions

Research design and conducted experiments: Shi M., Huang R. S., Guo F., Zhou L., Ma L., and Fu P. Performed data analysis: Shi M., Guo F., Li L. Z., Feng Y. Y., and Wei Z. J. Contributed to the writing of the manuscript and English revision: Shi M., Huang R. S., Li L. Z., Feng Y. Y., Ma L., Zhou L., and Fu P.

## Conflicts of interest

There are no conflicts to declare.

## Supplementary Material
